# Genome-Wide Identification and Hormone Response Analysis of the COBL Gene Family in Barley

**DOI:** 10.3390/genes15050612

**Published:** 2024-05-11

**Authors:** Panrong Ren, Liang Ma, Wei Bao, Jie Wang

**Affiliations:** 1 School of Agriculture and Bioengineering, Longdong University, Qingyang 745000, China; sakuramaliang@gmail.com (L.M.); 15010205598@163.com (W.B.); 2 College of Life and Environmental Sciences, Hangzhou Normal University, Hangzhou 311121, China; 18627934387@163.com

**Keywords:** barley, cellulose, COBRA-Like, genome-wide analysis

## Abstract

Barley (*Hordeum vulgare* L.), a diverse cereal crop, exhibits remarkable versatility in its applications, ranging from food and fodder to industrial uses. The content of cellulose in barley is significantly influenced by the *COBRA* genes, which encode the plant glycosylphosphatidylinositol (GPI)-anchored protein (GAP) that plays a pivotal role in the deposition of cellulose within the cell wall. The COBL (COBRA-Like) gene family has been discovered across numerous species, yet the specific members of this family in barley remain undetermined. In this study, we discovered 13 COBL genes within the barley genome using bioinformatics methods, subcellular localization, and protein structure analysis, finding that most of the barley COBL proteins have a signal peptide structure and are localized on the plasma membrane. Simultaneously, we constructed a phylogenetic tree and undertook a comprehensive analysis of the evolutionary relationships. Other characteristics of *HvCOBL* family members, including intraspecific collinearity, gene structure, conserved motifs, and cis-acting elements, were thoroughly characterized in detail. The assessment of *HvCOBL* gene expression in barley under various hormone treatments was conducted through qRT-PCR analysis, revealing jasmonic acid (JA) as the predominant hormonal regulator of *HvCOBL* gene expression. In summary, this study comprehensively identified and analyzed the barley *COBL* gene family, aiming to provide basic information for exploring the members of the *HvCOBL* gene family and to propose directions for further research.

## 1. Introduction

COBRA is a crucial glycosylphosphatidylinositol (GP-I) anchor protein (GAP), which plays a vital role in plant cellulose synthesis and significantly impacts the mechanical strength of plants. COBL (COBRA-Like) specifically localizes on the exterior surface of the plant plasma membrane, receives signals from the cell wall, and promptly transmits them to the plasma membrane [1]. There are four conserved domains contained within the COBRA protein, as follows: (1) the N-terminal protein-targeting domain, which can localize proteins in the endoplasmic reticulum. (2) The carbohydrate-binding motif domain, the main region interacting with cellulose [2]. (3) The conserved CCVS domain, which is thought to be involved in disulfide bond formation or metal ion binding [3]. (4) The hydrophobic C-terminal domain, where the connection region of anchoring proteins that promote the transport of certain proteins to the cell wall exist [4].

The *COBL* gene was initially discovered in *Arabidopsis thaliana*, and *COBL* mutation causes abnormal root cell expansion [5]. Subsequent research has shown that *COBL* is crucial for the orientation of cellulose microfibrils and for anisotropic expansion during plant morphogenesis [6]. Absence of the COBL protein in *A. thaliana* typically results in a reduction in the thickness of the secondary wall and a decrease in the content of cellulose [7,8]. Mutation of *COBL10* in *A. thaliana* results in gametophytic male sterility, indicating the significance of *COBL10* in guiding growth and directional perception within the female reproductive tract of pollen tubes as well as its crucial role in intercellular communication within plants [9].

The *COBL* genes were originally identified as “brittle stalk” genes in *O. sativa* and *Z. mays*. The “*Os brittle culm 1*” mutant and the “*Zm brittle stalk 2*” mutant all exhibit the characteristics of reduced cellulose content and cell wall thickness, and the mechanical strength of their stems is greatly compromised [10,11]. COBL plays a crucial role in the deposition of cellulose within the mucilage secretory cells of the seed coat in angiosperms. It serves as a vital component in the production of seed mucilage, thereby contributing significantly to the adaptation of plants to their diverse environments [12]. Notably, COBL also plays a role in plant fruit development. The *COBL* gene found in tomatoes, which plays a major role in the early development of fruit cell walls, has been shown to enhance the hardness and prolong the storage time of tomato fruits in overexpression transgenic lines [13].

Cellulose, the primary constituent of plant cell walls, serves as the foundation for the spatial structure of plant tissues and confers mechanical strength to plants, ensuring the orderly development of plants while bolstering their resilience against environmental stress. Barley is a cereal crop that thrives at elevated altitudes, serving as a dual-purpose resource for both human consumption and animal feeding. The abundant cellulose in barley is of utmost importance, crucial in averting lodging, maintaining consistent yields, and acting as a precious industrial raw material [14].

In barley (*H. vulgare* L.), the influence of cellulose synthase and cellulose synthase-like enzymes on the roots, stems, and associated biological reaction processes, achieved through their regulatory role in cellulose content, has been elucidated [15,16,17]. However, although one COBL gene associated with cellulose synthesis was pinpointed through genome-wide association studies (GWAS) conducted to investigate cellulose content in barley [18], the information on *COBL* family genes and their effect on cellulose in barley remains unclear. In this study, we performed a comprehensive genome-wide identification of the *COBL* gene family in the barley genome, resulting in the discovery of 13 candidate *COBL* genes. Subsequently, we conducted detailed studies encompassing various aspects such as domains, motifs, gene structure, phylogenetic relationships, and cis elements. In addition, signal peptide, transmembrane helix, and subcellular localization analyses indicate that most *COBLs* are membrane proteins in barley. Quantitative reverse-transcription PCR (qRT-PCR) analysis was employed to further validate the expression patterns of *HvCOBL* genes across various organs and under different abiotic stress conditions. This investigation provides a robust foundation for future studies aimed at elucidating the functional roles of *COBL* genes in barley.

## 2. Results

### 2.1. Identification of the COBL Gene Family in the Barley Genome

HvCOBL family genes are distributed across six barley chromosomes except chromosome 1. ExPASy online software was used to predict the physicochemical properties of the HvCOBL family proteins. The lengths of the 13 identified HvCOBL proteins ranged from 222 to 673 aa, and their molecular weights ranged from 23.96 to 74.57 kDa. The predicted isoelectric point ranged from 5.02 (HvCOBL1) to 9.02 (HvCOBL12), and the instability index ranged from 27.41 (HvCOBL5) to 57.86 (HvCOBL4). The hydrophilicity values of all HvCOBL proteins except HvCOBL1 are less than 0, indicating that most of them are hydrophilic proteins. The Aliphatic index, which indicates the solubility and stability of proteins, ranged from 64.52 (HvCOBL12) to 79.71 (HvCOBL1). In addition, the size, location, and orientation of the HvCOBL family genes on chromosomes are shown in Table 1, the gene IDs corresponding to the gene name are listed in Table S3, and the CDS sequences and protein sequences of the HCOBL genes are shown in Table S4.

### 2.2. Chromosomal Location and Collinearity Analysis of COBL Family Genes in Barley

**Figure 1:**
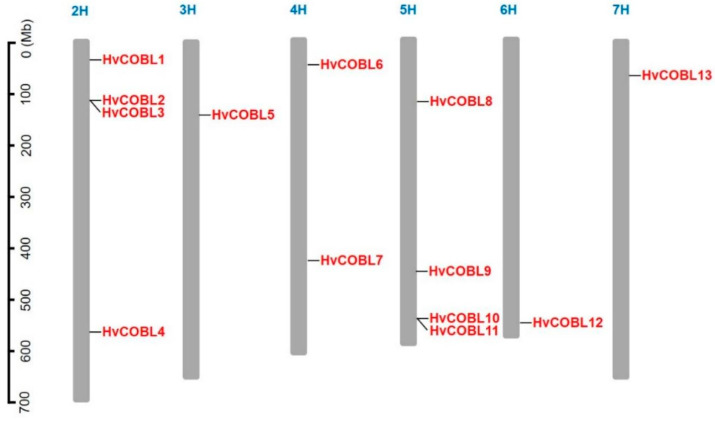
The position of *HvCOBL* gene family members on chromosomes. The scale on the left is used to indicate the length of chromosomes. The *HvCOBL* gene family members on the chromosomes (gray bar) are marked in red, and the blue numbers represent chromosome numbers.

The identified HvCOBL family gene members are distributed on six chromosomes (2H–7H) in barley, including four *COBL* genes on chromosomes 2 and 5, two COBL genes on chromosome 4, and only one *COBL* gene each on chromosomes 3, 6, and 7. As shown in Figure 1, the HvCOBL family genes are, mostly, relatively close to the telomeric region of the chromosome. Furthermore, the four HvCOBL gene pairs situated on chromosomes 2 and 5, respectively, exhibit a remarkable proximity to each other, hinting at a potential genetic linkage among them.

**Figure 2:**
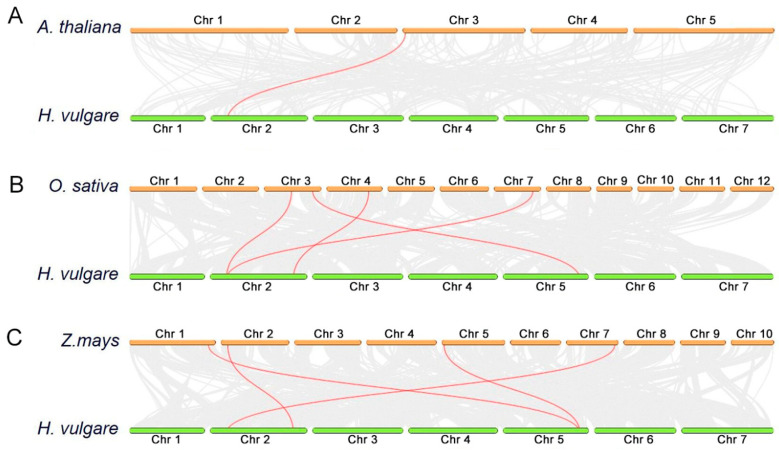
Interspecific collinearity relationship between *HvCOBL* gene family members and *A. thaliana* (**A**), *O. sativa* (**B**), and *Z. mays* (**C**). The chromosomes of *H. vulgare* are marked with green. The chromosome number is marked above the chromosome. The collinear relationships between the *COBL* gene family members of different species and the *HvCOBL* gene family members are connected by red lines.The gray line links all genes that exhibit collinear relationships between different species, excluding the COBL family.

To clarify the collinear relationship between COBL family gene species, we performed collinearity analysis using the genomes of *A. thaliana*, *Zea mays*, and *Oryza sativa*, as well as the barley genome. Only one collinearity associated with *COBL* genes was detected between barley and *A. thaliana* (Figure 2A), while four were identified with *O. sativa* (Figure 2B) or *Z. mays* (Figure 2C). This result indicates that compared to *A. thaliana*, the COBL family genes of barley, maize, and rice, which are also gramineous plants, are more closely related to the evolutionary process. Furthermore, it seems that the common ancestor of *O. sativa* and *H. vulgar* have one copy (*HvCOBL2* in the Chr2 in *H. vulgar*) that after separation originated two copies in *O. sativa* by duplication (one in Chr3 and the other in Chr7); *HvCOBL11* (in the Chr5 in *H. vulgar*) appears to exhibit a comparable phenomenon, with the homologous genes of maize undergoing separation during evolution, resulting in the production of two copies located on Chr1 and Chr5.

### 2.3. Phylogenetic Relationship Analysis of COBL Proteins

**Figure 3:**
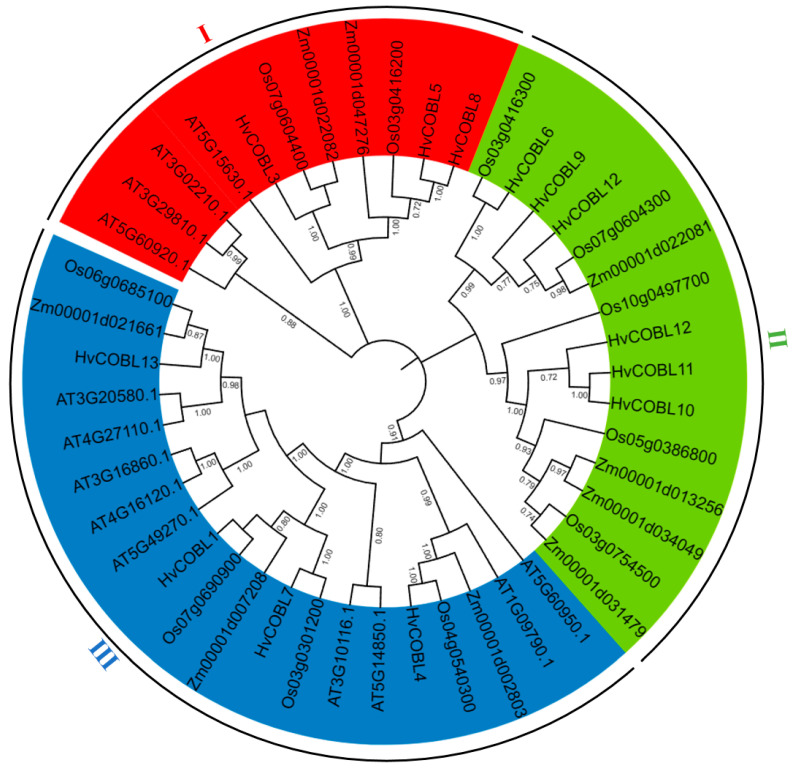
The phylogenetic analysis of COBL family proteins in four species. The neighbor-joining tree was constructed from the protein sequences of COBLs using MEGA7 with 1000 bootstrap copies. Bootstrap analysis values over 0.70 that are displayed in the tree indicate robust nodes. The different colors represent four different subfamilies of the *COBL* gene family.

To investigate the phylogenetic relationships between the COBL proteins, we constructed a phylogenetic tree using COBL protein sequences from different species, including *A. thaliana* (13 proteins), *Z. mays* (12 proteins), *O. sativa* (14 proteins), and *H. vulgare* (13 proteins), respectively. All the protein sequences are shown in Table S5. As shown in Figure 3, the phylogenetic tree was divided into three subfamilies. There are 11 COBL proteins belonging to subfamily I, including 3 HvCOBL proteins (HvCOBL3, HvCOBL5, HvCOBL8), 2 OsCOBL proteins, 4 AtCOBL proteins, and 2 ZmCOBL proteins; 15 COBL proteins belong to subfamily II, including 6 HvCOBL proteins (HvCOBL2, HvCOBL6, HvCOBL9, HvCOBL10, HvCOBL11, HvCOBL12), 5 OsCOBL proteins, and 4 ZmCOBL proteins; and 20 COBL proteins belong to subfamily III, including 4 HvCOBL proteins (HvCOBL1, HvCOBL4, HvCOBL7, HvCOBL13), 4 OsCOBL proteins, 3 ZmCOBL proteins, and 9 AtCOBL proteins. 

### 2.4. Analyses of Locations, Structures, and Conserved Motifs of HvCOBL Genes

**Figure 4:**
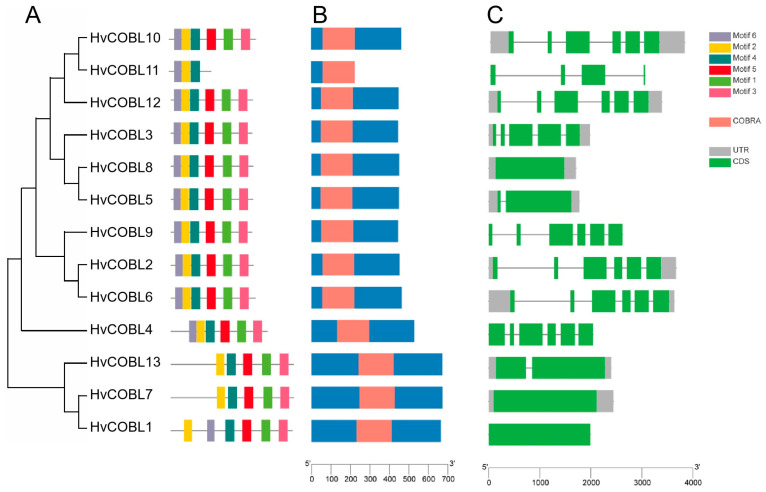
Gene structure, domain, and conserved motifs in *HvCOBL* gene family members. (**A**) Phylogenetic tree of *HvCOBL* gene family members. Distribution of conserved motifs of *HvCOBL* genes. The different colored boxes indicate different conserved motifs. (**B**) Distribution of COBRA domain. The orange box indicates the location of the COBRA domain on the peptide chain. (**C**) Distribution of UTRs and CDSs of *HvCOBL* gene family members. The gray boxes represent UTRs, the green boxes represent exons, and the gray lines represent introns. The axes at the bottom are used to compare the lengths of different genes and proteins.

To further reveal the structural characteristics of the *HvCOBL* family genes, we analyzed the conserved motifs of the 13 *HvCOBL* genes using the MEME online tool. All 13 HvCOBL proteins shared two common motifs (motif 2 and motif 4) (Figure 4A), indicating that these two motifs are important for HvCOBL proteins. In addition, motif 5 (red boxes), which contains the CCVS conserved motif of the COBRA protein, is a key amino acid region for function. The exon–intron structures of *HvCOBL* genes were analyzed by using the CDS and genome sequences. As shown in Figure 4, among all the COBL family gene members in barley, there are up to six exons with five introns (*HvCOBL2*, *HvCOBL*4, *HvCOBL*6, *HvCOBL*9, *HvCOBL*10, *HvCOBL*12) for a gene and as few as one with no intron (*HvCOBL* 1, *HvCOBL* 8). Indeed, introns serve to elongate genes and enhance the frequency of recombination among them, thereby playing a pivotal role in promoting the evolution of a species. Among the *COBL* family genes in barley, five genes (*HvCOBL2*, *HvCOBL*6, *HvCOBL*10, *HvCOBL*11, *HvCOBL*12) possess relatively longer introns, followed by another five genes (*HvCOBL3*, *HvCOBL*4, *HvCOBL*5, *HvCOBL*9, *HvCOBL*13) with shorter but still present introns. Notably, three genes (*HvCOBL* 1, *HvCOBL* 7, *HvCOBL* 8) completely lost their introns during the process of evolution.

### 2.5. Prediction of Signal Peptide, TMHs, and Subcellular Localization of HvCOBL Family Proteins

To acquire information on the signal peptide and transmembrane domain of HvCOBL family proteins, we employed the amino acid sequences of HvCOBL proteins to predict them using the SignalP 4.0 and TMHMM-2.0 websites, respectively. The signal peptide prediction and TMHMM results of the HvCOBL proteins are shown in Table 2. The predicted score values strongly support that all the HvCOBL members contain N-terminal signal peptides with the exception of HvCOBL4 (Table S6). The TMHMM prediction results reveal that among the 13 HvCOBL proteins, 7 members do not possess transmembrane helices, while 4 HvCOBL proteins are predicted to have one transmembrane helix, and 2 HvCOBL proteins are predicted to exhibit two transmembrane helices. The positions of the transmembrane helices on the peptide chain of HvCOBL protein are shown in Table S7. In addition, the hydrophobicity of each amino acid in the HcCOBL protein sequences was predicted using an online tool, and the results are displayed in Table S8.

The presence of signal peptides and transmembrane helices exerts an influence on the subcellular distribution of proteins. To further elucidate the subcellular localization patterns of the HvCOBL family proteins, we employed three online bioinformatics tools (WOLF PSORT, Plant-mPLoc, and CELLO) to predict the subcellular localization of HvCOBL proteins in barley. The results of the subcellular localization predictions from various subcellular localization prediction tools exhibit consistent trends, and the predicted scores suggest that most members of HvCOBL are likely to localize in the plasma membrane, lysosomes, and extracellular space (Table S9).

### 2.6. Analysis of Cis-Acting Elements of HvCOBL Promoter

**Figure 5:**
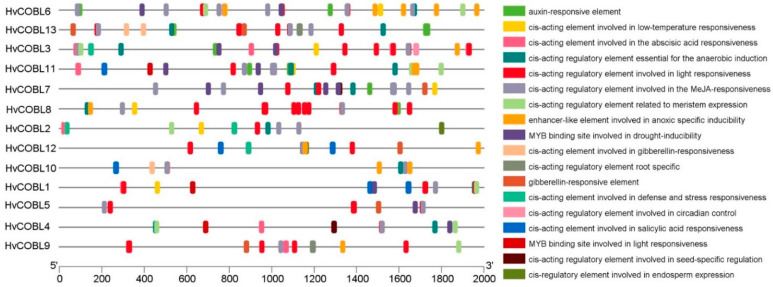
Analysis of cis-acting elements in the *HvCOBL* gene family promoter. The distribution of cis-acting elements in the *HvCOBL* promoter. Different colors and boxes represent different types of cis-acting elements. The scale at the bottom indicates the length of the promoter sequence (0~2000 bp) and the location of the cis-acting elements.

Cis-elements on gene promoters, which are important for gene transcription, serve as binding sites for transcription factors, enabling the activation or repression of gene expression patterns during plant growth, development, and adaptation to external environmental stresses. To investigate the expression profiles of *HvCOBL* family genes, a 2000 bp sequence upstream of each gene was used to predict cis-acting elements through the PlantCARE website. As shown in Figure 5, we found that a total of 36 cis-acting elements involved in hormone response were predicted in the promoters of 13 *HvCOBL* genes, including 10 auxin-responsive elements, 44 abscisic acid-responsive elements, 64 MeJA-responsive elements, 9 gibberellin-responsive elements, and 6 salicylic acid-responsive elements. Among the 13 *HvCOBL* genes, all of their promoters contain more than one MeJA response element, indicating that the expression of COBL genes in barley is closely related to jasmonic acid. Furthermore, the promoter of the HvCOBL gene contains numerous MYB elements that are associated with drought response, thereby implying that the expression of the *HvCOBL* genes may be modulated by drought stress. Details of cis-acting elements in the promoter region of *HvCOBL* genes shown in Table S10.

### 2.7. Expression of HvCOBL Genes under Different Plant Hormone Treatments

**Figure 6:**
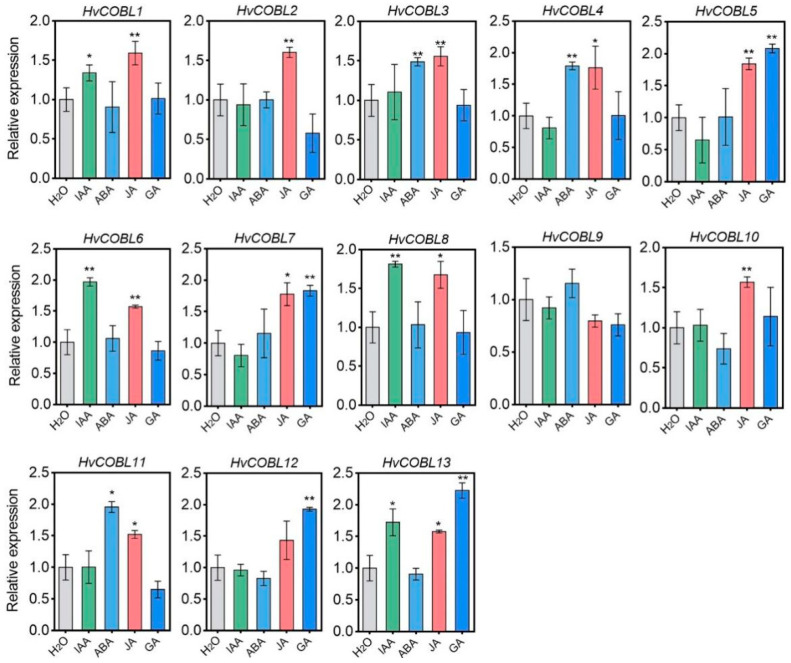
Analysis of gene expression levels of *HvCOBL* gene family members in barley seedlings under different plant hormone treatments (IAA, ABA, JA, GA). Data are the means ± standard error (*n* = 3). Asterisks indicate the *p* value determined using a Student’s *t* test (* *p* < 0.05, ** *p* < 0.01).

Plant hormones, including auxins and abscisic acid, etc., play a pivotal role in regulating plant growth, development, and stress response mechanisms. The promoter sequences of *HvCOBL* gene family members encompass numerous cis-acting elements that are associated with plant hormone response, indicating that hormones potentially influence the expression of *HvCOBL* genes. To delve into the impact of plant hormones on *HvCOBL* gene expression, we administered diverse hormones to barley seedlings at the four-leaf stage and subsequently employed RT-qPCR to assess the expression levels of *HvCOBL* genes. AS shown in Figure 6, the expression of all the *HvCOBL* family members is induced by jasmonic acid (JA), albeit the expression level of *HvCOBL12* did not attain statistical significance. This observation suggests that JA plays a pivotal regulatory role in plant cellulose accumulation when compared to other plant hormones. Furthermore, *HvCOBL1*, *HvCOBL6*, *HvCOBL8*, and *HvCOBL13* were strongly upregulated by indole-3-acetic acid (IAA); *HvCOBL5*, *HvCOBL7*, *HvCOBL12*, and *HvCOBL13* exhibited sensitivity to gibberellin signaling; and the expressions of *HvCOBL3*, *HvCOBL4*, and *HvCOBL11* were associated with abscisic acid (ABA). These results suggest that plant hormones have great potential in regulating plant cellulose content. 

## 3. Discussion

### 3.1. Functions of Cellulose in Stem Development and Stress Responses

Cellulose, an essential morphogenic polysaccharide and the main component of plant cell walls, determines the size of cells, provides structural support for the plant body, protects cells from pathogens, and serves as a communication bridge between the apoplast and symplast [19]. Owing to their primary composition of carbohydrates, the profuse abundance of cell walls establishes them as the largest carbon sink on Earth. Once cellular growth ceases, specific cell types deposit thick secondary cell walls (SCWs) as a means of bolstering a cell’s resistance to both mechanical and biotic stresses [20]. The crucial role of COBL family members in cellulose synthesis has been elucidated in multiple species, and the content of cellulose determines the stem breakage resistance of plants [11,21,22]. The drought resistance of plants is influenced by the opening and closing of stomata, which are mediated by guard cells. The dynamic reorganization of cellulose microfibrils within these guard cells occurs during stomatal movement, and the levels of cellulose and xyloglucan have a direct impact on the kinetic properties of guard cells [23]. The *A. thaliana* COBL7 protein and COBL8 protein jointly participate in the processes of cell division and stomatal development, influencing the formation of stomatal pores and the morphological development of stomata through the regulation of cellulose deposition and cell wall modification [24]. Cellulose plays a pivotal role in plants’ responses to gravity, while brassinolide regulates the content of cellulose and mannan in response to gravity, serving as one of the key mechanisms underlying plants’ gravitational responsiveness [25]. Additionally, overexpression of the *COBL* gene significantly boosts cellulose synthesis and deposition, effectively promoting plant cell elongation and thickening and, consequently, enhancing the overall biomass of a plant. This approach offers a promising avenue for the improvement of crop yield and quality [26]. The rice *DROT1* gene encodes a COBL protein that enhances drought tolerance in rice by modulating cell wall structure, elevating cellulose content, and preserving cellulose crystallinity [27]. Additionally, members of the *COBL* gene family exhibit responsiveness to abiotic stimuli, including heat and cold [28,29]. The analysis of the promoter sequences of HvCOBL gene family members in barley revealed the presence of anaerobic response elements associated with waterlogging stress, as well as drought stress response elements (Figure 5); this indicates that the accumulation of cellulose in barley may be one of the key factors in its response to drought or waterlogging.

### 3.2. COBL Family Genes Involved in Cellulose Synthesis

The COBL family proteins, although not direct cellulose synthases, exert a direct influence on cellulose content within the plant body and possess the ability to regulate the orientation of cellulose microfibrils [6,30]. Cellulose synthase is composed of cellulose synthase subunit A (*CesA*) and cellulose-like synthase (*Csl*), which are encoded by the cellulose synthase family genes, and these catalytic processes occur in the Golgi apparatus and plasma membrane [31,32,33]. Unlike hemicellulose and pectin, which are assembled in the Golgi apparatus and subsequently exported to the cytoplasm via exocytosis, cellulose is produced on the plasma membrane [34]. The expression of *COBL* family genes is observed throughout the developmental stages of both dicots and monocots, and this expression can be modulated by environmental conditions [3,35]. Brittle Culm1 (BC1), a COB-like protein, possesses a carbohydrate-binding module that specifically interacts with crystalline cellulose and exhibits the function of altering microfibril crystallinity, suggesting that the COBL protein participates in the assembly of cellulose microfibrils by modulating the crystallinity of cellulose [4]. The cellulose synthase complex (*CSC*) serves as the primary catalyst in the biosynthesis of cellulose within plant cell walls and comprises numerous cellulose synthase (CesA) subunits. Despite ongoing research, there remains no consensus on the precise assembly model of the CSC. While the COBL protein is essential for maintaining the correct orientation of microfibrils, the intricacies of its interaction with the CSC complex remain an area of active investigation, awaiting further elucidation. As a pivotal growth regulator for plants, hormones exert a crucial influence on the expression of the *HvCOBL* genes in barley. Cis-acting elements pertaining to diverse auxin responses, encompassing IAA, ABA, JA, and GA, were discovered on the *HvCOBL* gene’s promoter (Figure 5). Subsequently, qRT-PCR validation confirmed the stimulatory impact of hormones on HvCOBL gene expression, with jasmonic acid emerging as a particularly significant hormone in this regard.

### 3.3. Regulating Cellulose Content to Assist Barley Production

Barley, a crucial grain and forage crop, finds extensive application in the brewing industry and biomass energy production, leading to a gradual expansion of its cultivation area across the globe [36]. The effect of cellulose content on barley production is profound. On one hand, abundant cellulose provides the necessary structural support for barley, enhancing its lodging resistance and adaptability to adverse conditions such as drought and cold, thus positively contributing to the growth and yield of barley. On the other hand, the regulation of cellulose content can also affect the quality of barley, including protein and fat content [37,38]. By regulating the synthesis and decomposition of cellulose, it is possible to influence the growth rate and morphology of barley, ultimately optimizing both its yield and quality. As an illustration, during the tillering stage, the synthesis of cellulose plays a crucial role in strengthening the cell wall, thereby fostering the formation and healthy growth of tillers. As a feed source, a reduced cellulose content in barley is preferred to facilitate the utilization of the entire plant for silage. The hay derived from this process boasts a comparably high protein content alongside a lower cellulose content, rendering its overall nutritional value superior to that of other crops like rice and corn. Nevertheless, for ruminants such as cattle and sheep, a moderate increase in cellulose content is essential to sustain the healthy functioning of their digestive systems. Cellulose can be used in industrial production, as pulp production, and in textile raw materials; it can be converted into biomass fuel to replace traditional fossil energy, which is a new direction for the development of renewable energy.

## 4. Materials and Methods

### 4.1. Identification of COBL Genes in Barley

The genome sequences of *H. vulgare* were obtained from the EnsemblPlants database (https://plants.ensembl.org/index.html, accessed on 2 January 2024). The published protein sequence of *A. thaliana* COBL family members were downloaded from the NCBI database (http://www.ncbi.nlm.nih.gov/, accessed on 2 January 2024). The HMM file corresponding to the COBL domain (PF04833) was downloaded from the Pfam protein family database (https://www.ebi.ac.uk/interpro/, accessed on 2 January 2024). Possible COBL family members were obtained according the COBL domain (PF04833) by using the HMM search function of ToolKit Biologists Tools (TBtools, v2.086) software (https://github.com/CJ-Chen/TBtools, accessed on 2 January 2024). In detail, we blasted the genome database of barley using the COBL protein sequences of *A. thaliana*, *O. sativa*, and *Z. mays*, and took the intersection of the results to verify the comparison with the Hidden Markov Model (HMM) (PF04833) to ensure that the identified genes contain the CORBA domain. The gene accession numbers, coding sequence lengths, and amino acid numbers were derived from the rice genome database, and the sequence information of the COBL candidate genes was obtained from Phytozome (https://phytozome-next.jgi.doe.gov, accessed on 2 January 2024). The molecular weight and isoelectric point were obtained from Expasy (http://web.expasy.org/cgi-bin/protaparam/protparam, accessed on 2 January 2024).

### 4.2. Phylogenetic Analysis and Classification of HvCOBL Genes in Barley

The COBL family protein sequences of *A. thaliana*, *Z. mays*, and *O. sativa* were downloaded from the Phytozome database (https://phytozome-next.jgi.doe.gov/, accessed on 5 January 2024), and the multiple amino acid sequence alignment of the *HvCOBL* proteins was performed using the ClustalW method implemented in Molecular Evolutionary Genetic Analysis software (MEGA 7). The phylogenetic tree was constructed by using MEGA7 with a bootstrap of 1000 replications using the neighbor-joining (NJ) method. The generated tree was displayed using the iTOL website (https://itol.embl.de/upload.cgi, accessed on 5 January 2024).

### 4.3. Collinearity Analysis and Chromosomal Mapping of the HvCOBL Family Genes

The annotation information and the whole-genome protein sequences of *A. thaliana*, *Z. mays*, *O. sativa*, and *H. vulgare* were obtained from the EnsemblPlants database (https://plants.ensembl.org/index.html, accessed on 5 January 2024). The collinearity relationships of the COBL genes between *H. vulgare*, *Z. mays*, *O. sativa* and *A. thaliana* genomes were determined and visualized by using the Multiple Collinearity Scan toolkit (MCScanX) of TBtools software with default parameters. The physical positions of the *HvCOBL* genes on the 7 chromosomes of the barley genome were determined and visualized using the TBtools software according to the genome annotation file.

### 4.4. Gene Structure, Domain Analysis, Motif Analysis, and Cis-Regulatory Analysis

The whole-genome sequences and CDS sequences of *HvCOBL* genes were downloaded and used for gene structure analysis with TBtools software. The motif of the HvCOBL family protein was analyzed by using the MEME website (http://meme-suite.org/tools/meme, accessed on 6 January 2024). The domain of the HvCOBL family protein was analyzed by using Batch CD-Search (https://www.ncbi.nlm.nih.gov/Structure/bwrpsb/bwrpsb.cgi, accessed on 6 January 2024). The promoter sequences 2000 bp upstream of the ATG start codon of the *HvCOBL* genes were extracted and submitted to Plant CARE (http://bioinformatics.psb.ugent.be/webtools/plantcare/html/, accessed on 10 January 2024) for cis-element analysis. The type and quantity of cis-elements were visually represented through a heat map generated using the TBtools software.

### 4.5. Protein Three-Dimensional Structure, Signal Peptide, and Transmembrane Helices Prediction of HvCOBL Proteins

The prediction of transmembrane helices in the HvCOBL proteins was performed using online software TMHMM-2.0 (https://services.healthtech.dtu.dk/services/TMHMM-2.0/, accessed on 8 January 2024). Prediction of the signal peptides in the HvCOBL proteins was performed using the online software SignalP-4.1 (https://services.healthtech.dtu.dk/services/SignalP-4.1/, accessed on 8 January 2024). Detailed results of the signal peptide and transmembrane helices of the HvCOBL proteins are shown in Tables S6 and S7. The hydrophobicity of each amino acid in the HcCOBL protein sequences was predicted using an online tool (https://www.novopro.cn/tools/protein-hydrophilicity-plot.html, accessed on 8 January 2024).

### 4.6. Plant Treatment and Quantitative RT-PCR

Barley seeds were germinated at 25 °C for 60 h and were then transplanted into soil and grown to the four-leaf stage under 16 h light (1000 ± 100 µmol·m^−2^·s^−1^)/8 h dark at 25 °C. For exogenous hormone treatment, four-leaf stage seedlings were investigated in separate 10 mM hormone solutions (specifically, IAA, ABA, JAor SA) at 25 °C for 6 h. Total RNA was extracted from the leaves of the barley using RNAiso Plus (Takara, Japan). About 0.4 g of basal internodes from the leaves were ground into powder in liquid nitrogen and transferred to 800 μL of RNAiso Plus. The final extracted RNA was dissolved in 25 μL of DEPC water. Total RNA (1.5 μg) was used to synthesize cDNA, and dilutions of it were used for real-time RT-PCR. The expression levels of the tested genes, *HvCOBL1* to *HvCOBL13,* were all normalized to *HvActin* (a constitutively expressed gene in barley). The primers and *HvCOBLs* are listed in Table S2. Gene expression levels were calculated by using the formula (E*_HvActin_*)^CT^*_HvActin_*/(E*_Target_*)^CT^*_Target_* [39].

## Figures and Tables

**Table 1:** *HvCOBL* gene family gene and protein properties.

Gene Name	Chr	Gene Location (from to)		Size	Number of Amino Acids	Mw (KD)	pI	Instability Index	Aliphatic Index	GRAVY
*HvCOBL1*	2	30,152,814	30,154,812	1998	665	71.02	5.02	39.35	79.71	0.039
*HvCOBL2*	2	11,084,0647	110,844,319	3672	452	50.35	8.23	33.83	74.25	−0.208
*HvCOBL3*	2	110,845,455	110,847,441	1986	444	48.93	8.96	32.58	73.13	−0.111
*HvCOBL4*	2	572,266,296	572,268,346	2050	528	58.99	7.14	57.86	75.68	−0.216
*HvCOBL5*	3	14,401,920	14,403,700	1780	449	49.2	8.86	27.41	74.32	−0.112
*HvCOBL6*	4	47,365,044	47,368,680	3636	463	51.25	8.87	32.07	78.75	−0.097
*HvCOBL7*	4	438,450,414	438,452,859	2445	673	72.2	6.2	33.56	79.48	−0.034
*HvCOBL8*	5	126,154,671	126,156,383	1712	450	49.55	8.86	31.2	74.96	−0.1
*HvCOBL9*	5	458,128,527	458,131,155	2628	444	49.55	8.65	36.79	76.82	−0.161
*HvCOBL10*	5	528,702,303	528,706,060	3757	457	50.74	8.97	39.28	68.32	−0.208
*HvCOBL11*	5	528,808,215	528,811,208	2993	222	23.96	8.97	40.74	73.87	−0.063
*HvCOBL12*	6	548,235,850	548,239,243	3393	420	47.45	9.02	33.69	64.52	−0.242
*HvCOBL13*	7	51,952,323	51,954,724	2401	672	74.57	8.93	43.76	71.03	−0.3

GRAVY value is a parameter that evaluates the overall hydrophobicity of a protein.

**Table 2:** Prediction of signal peptides and TMHs of HvCOBL family proteins.

Protein Name	Signal Peptide Prediction			TMHs Prediction	
	C-Score	S-Score	Signal Peptide	TMHs Number	TMhelix Location
HvCOBL1	0.856	0.978	YES	1	84~106
HvCOBL2	0.826	0.994	YES	0	-
HvCOBL3	0.67	0.972	YES	0	-
HvCOBL4	0.155	0.186	NO	2	5~27, 434~456
HvCOBL5	0.834	0.967	YES	0	-
HvCOBL6	0.869	0.995	YES	0	-
HvCOBL7	0.78	0.954	YES	2	2~24, 649~671
HvCOBL8	0.865	0.96	YES	0	-
HvCOBL9	0.737	0.981	YES	1	642~664
HvCOBL10	0.748	0.955	YES	0	-
HvCOBL11	0.777	0.937	YES	0	-
HvCOBL12	0.796	0.977	YES	1	7~24
HvCOBL13	0.837	0.968	YES	1	7~24

Note: C-score: cleavage site score; S-score: signal peptide score; TMhelix: position of transmembrane helix.

## Data Availability

All relevant data can be found within the manuscript and its Supplementary Materials.

## Supplementary Materials

The following supporting information can be downloaded at: https://www.mdpi.com/article/10.3390/genes15050612/s1, Table S1: The list of abbreviations. Table S2: The list of primers used for qRT-PCR. Table S3: The list of Gene ID. Table S4: The list of proteins and CDs sequences of HvCOBL. Table S5: The list of protein sequences for constructing the phylogenetic tree. Table S6: The prediction of protein signal peptides. Table S7: The prediction of protein TMHs. Table S8: The Prediction of amino acid hydrophobicity of HvCOBL protein. Table S9: The prediction of subcellular localization of HvCOBL protein. Table S10: Information on cis-acting elements on the promoter.
